# Improving metabolic support during normothermic *ex-situ* heart perfusion

**DOI:** 10.3389/fcvm.2025.1687255

**Published:** 2026-01-05

**Authors:** Mitchell J. Wagner, Sanaz Hatami, Parham Hassanzadeh, Gopinath Sutendra, Jennifer Conway, Darren H. Freed

**Affiliations:** 1 Department of Surgery, University of Alberta, Edmonton, AB, Canada; 2 Department of Medicine, University of Alberta, Edmonton, AB, Canada; 3 Department of Pediatrics, University of Alberta, Edmonton, AB, Canada; 4 Department of Pediatric Cardiology, Stollery Children’s Hospital, Edmonton, AB, Canada; 5 Division of Cardiac Surgery, Department of Surgery, University of Alberta, Edmonton, AB, Canada; 6 Mazankowski Alberta Heart Institute, Edmonton, AB, Canada; 7 Alberta Transplant Institute, Edmonton, AB, Canada; 8 Canadian Donation and Transplantation Research Program, Edmonton, AB, Canada

**Keywords:** *ex-situ* heart perfusion, heart transplantation (HTx), cardiac metabolism, machine perfusion, organ preservation

## Abstract

*Ex-situ* heart perfusion (ESHP) is an innovative technology that has the power to greatly improve donor heart utilization and may eventually provide a platform for improvement of suboptimal hearts. However, its impact is limited by functional decline whilst on the platform, which is characterized by the development of oxidative stress and inflammation. Pathologic metabolism during normothermic ESHP may be an underlying factor in the development of such characteristics, however it is understudied within the context of machine perfusion. In the following review article, we discuss the limitations of the current metabolic substrate provision approach during ESHP (analogous to post-prandial glucose and insulin) from a mechanistic standpoint. We discuss alternative approaches and substrates that may be more conducive to physiologic preservation and recovery on the platform. We advocate for a support strategy mimicking fasting insulin and glucose, and alternative substrates such as free fatty acids and ketone bodies, which may be more adapted to the non-physiologic state encountered during ESHP. Throughout, we outline research gaps yet to be explored that would enable substrate provision approaches during machine perfusion of the donor heart to be further optimized.

## Introduction: heart failure, the need for *ex-situ* heart perfusion, and current challenges

Heart transplantation is the gold standard treatment for end-stage heart failure, an incurable condition that is on the rise globally (1). A recent estimate reported that the lifetime risk of acquiring heart failure has increased to roughly 1 out of every 4 people in the United States (2), representing a continued increase in the potential demand for heart transplantation. However, the amount of heart transplants performed is not keeping pace: recent reports from the International Society for Heart and Lung Transplant (ISHLT) show that though heart transplant volume across North America and Europe has climbed in recent years to roughly 6,000 per year, there are still roughly 7,000–8,000 on the waitlist in the USA alone at any given moment (3–5) Until this bottleneck is widened, individuals' severity of illness and risk of death on the waitlist will continue to be a pressing issue. A lack of organ preservation technology plays a large role in the estimated 28,000 life-saving organs discarded each year in the United States alone (6). The standard use of cold storage, while cheap and simple, limits preservation of the procured heart to 4–6 h, whereby longer periods are associated with increased prevalence of primary graft dysfunction post-transplant (7, 8). Therefore, innovation is necessary to increase supply through more efficient utilization of donor organs. Machine perfusion during transport is one way to tackle the ongoing need for donor organs.

*Ex-situ* heart perfusion (ESHP) describes the group of devices that enable machine perfusion of the donor heart during transport to the recipient site. Instead of cold storage, procured hearts are provided with metabolic needs using adjustable flow pumps, and can even simulate physiological afterload through the provision of working mode (9). ESHP may be carried out at hypothermia (4°C–10°C) or normothermia (37°C), with the latter being well suited as a platform to deliver semi-physiological preservation and enable therapeutic interventions, given that metabolism proceeds at physiological rates (10, 11). In the clinical setting, normothermic ESHP in the non-working mode by the TransMedics Organ Care System (OCS) Heart has been shown to lengthen preservation times with non-inferior outcomes (12). Therefore, ESHP can widen the bottleneck by facilitating longer range donor-recipient matching and increase utilization of procured organs. Herein, references to ESHP describe perfusion carried out at normothermic temperatures, unless stated otherwise.

Despite this potential, ESHP is currently limited by characteristic functional decline over the course of perfusion, which both limits the post-procurement preservation period and presents concerns about pathology at the level of the tissue (13, 14). The exact mechanism behind this process is unknown, however our group has been on the leading edge of investigation within this area. Our studies have revealed that functional decline during normothermic ESHP is associated with an intermingling of dysregulated metabolism, oxidative stress, and inflammation (15). Few studies have endeavored to understand the link between myocardial metabolism and these processes in the context of ESHP, and represent an opportunity to improve preservation. In the following review, we provide a discussion of the mechanistic drawbacks of current substrate provision approaches during ESHP, and advocate for alternative metabolic support strategies with the potential to improve preservation on the platform via the current understanding of pathophysiology during ESHP.

## Pathophysiology during ESHP: entangled oxidative stress, inflammation, and metabolism

**Figure 1:**
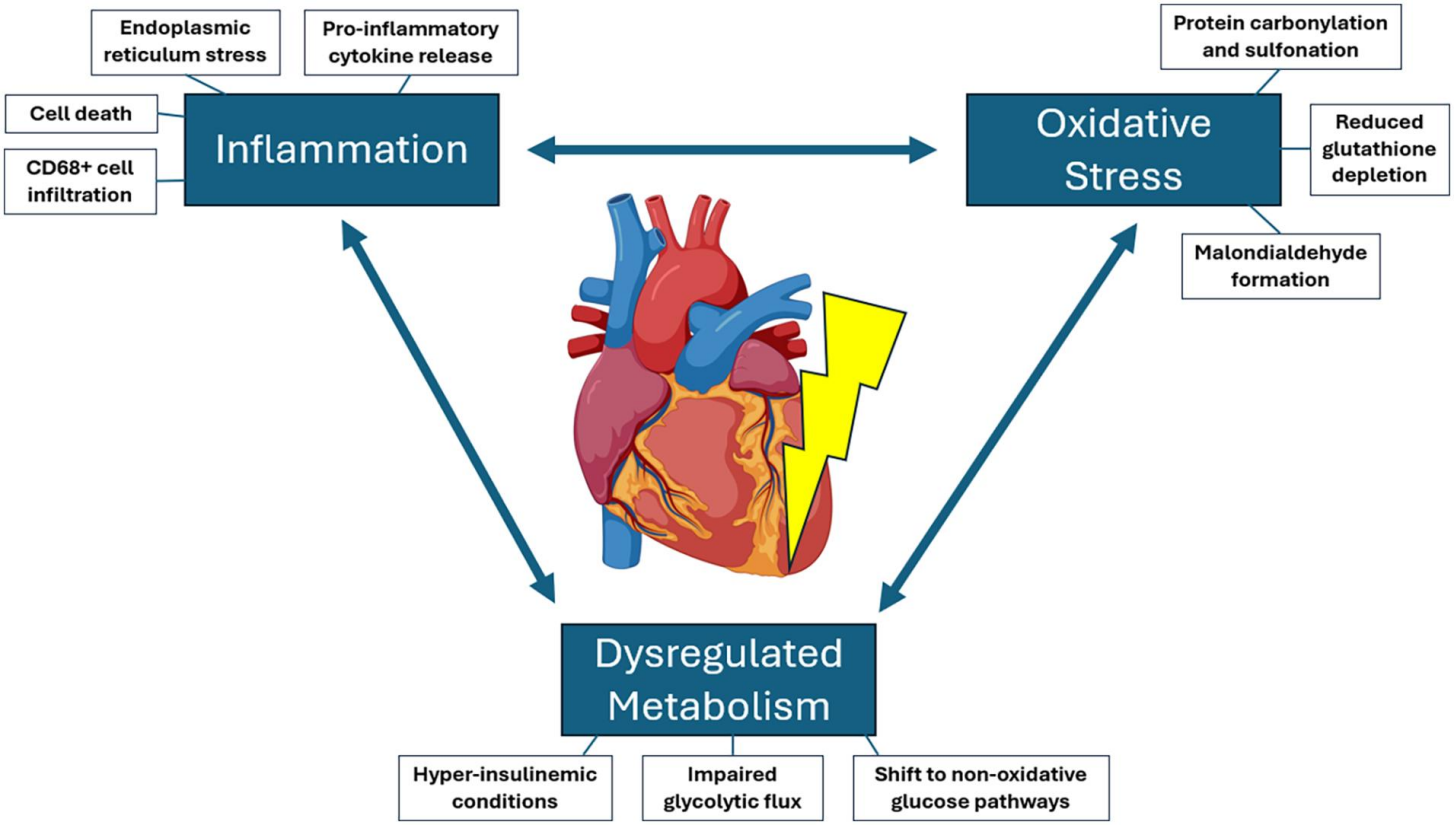
Overview of pathophysiologic manifestations during ESHP. Pathology to the heart on the ESHP apparatus appears to be underpinned by the intermingling of inflammation, oxidative stress, and dysregulated metabolism, which each influence each other during ESHP. Heart clipart courtesy of BioRender (https://www.biorender.com/).

Understanding the molecular mechanisms that underpin functional decline and its underlying pathophysiology during ESHP can unlock targeted support strategies to affect longer and more efficacious preservation, such that procurement, transport, and implantation of the donor heart is effectively seamless. While it is understood that preservation of the donor heart with ESHP is associated with an intermingling of oxidative stress, inflammation, and metabolic dysregulation (14, 16) (Figure 1), what mediates these phenomena is still under investigation. Our research group has demonstrated that a variety of oxidative stress markers such as malondialdehyde (MDA), global carbonylation and sulfonation of proteins are increased, and the concentration of reduced glutathione is decreased during normothermic ESHP after 8–12 h (16). While such oxidative stress markers could be resultant from CD68 + cell infiltration of the myocardium observed during ESHP (17) (representative of macrophages, monocytes, and neutrophils), perfusion with a leuko-reduced perfusate resulted in neither a reprieve from decline in functional parameters, nor exacerbations in oxidative stress markers. In fact, we found that leuko-reduction resulted in increased TNF-α concentration by mid-perfusion, increased protein carbonylation, and worsened dP/dT minimum preservation by late perfusion (16). Additionally, working mode (WM) perfusion [which in comparison to non-working mode (NWM) has demonstrated improved functional preservation as well as reductions in inflammatory activation and oxidative stress] had a greater total leukocyte infiltrate compared to non-working mode perfusion (17).

We found from analysis of cell death following perfusion that the percentage of TUNEL positive cells (a marker for cell apoptosis) did increase following ESHP, however the overall percentage in all groups was less than 1.5% of cells. This is corroborated with other markers of apoptosis such as cleaved caspase-3, apoptosis inducing factor (AIF) and CCAAT-enhancer-binding protein homologous protein (CHOP) trending towards increases during ESHP, but generally not being significantly increased (13). It seems that the total amount of apoptotic cell death is not great enough to justify the degree of functional decline observed; though, a small amount of necrosis endured could contribute to the state of inflammation present. Regardless, these results suggest that during ESHP, oxidative stress occurs within the heart and initiates a small amount of cell death and inflammation, rather than the other way around. Immune cells may perhaps even play a protective role during ESHP, given that their depletion from the perfusate displayed worsened biochemical and functional parameters. Of course, ischemia-reperfusion injury will contribute to the development of oxidative stress once the organ is placed back on the ESHP circuit. However, given that functional decline continues to occur even once the heart is restabilized on the apparatus suggests that the environment of ESHP mediates a steady insult to the tissue, that may or may not be primed by ischemia-reperfusion injury.

### Supraphysiologic glucose and insulin provision as a contributor to ESHP pathophysiology

One can appreciate that donor organs, once implanted in the recipient, last for years as opposed to a matter of hours on a machine perfusion apparatus; therefore, ameliorating differences between the body and the ex-vivo environment of ESHP are likely to improve the efficacy and length of preservation. Studies of plasma cross-circulation with a live animal during machine perfusion have shown improved efficacy and length of preservation, though the mechanism of these effects is yet to be ascertained (18, 19). Metabolic support is one aspect of ESHP that demonstrates a considerable departure from the *in vivo* environment. For example, the current approach to substrate provision during clinical ESHP is to provide large doses of insulin and glucose, simulating the post-prandial (post-meal) period (9). Clinically approved TransMedics OCS Heart perfusion protocols call for the addition of 80 IU of insulin to the perfusate (12), constituting a final concentration of >50 IU/L, which is significantly greater than even physiological post-prandial serum insulin of up to 0.3 IU/L (20). Furthermore, it should be noted that the pharmacokinetics of hormonal provision during ESHP is yet to be characterized. Theoretically, one could surmise that due to a lack of renal dynamics and a significantly lower mass of tissue, and thus a lower number of available receptors to internalize circulating hormones, their potency is probably greater during ESHP as compared to the intact organism. Therefore, on top of supraphysiologic concentrations being provisioned, increased hormonal signalling of insulin would contribute to a metabolic outlay during ESHP similar to diabetic individuals, and perhaps may contribute to metabolic dysregulation and cardiac dysfunction in a similar fashion to that observed in diabetic cardiomyopathies (21). In contrast, it has been known for decades that caloric restriction without starvation provides an extension of lifespan for the intact organism, thought to be the result of improved redox balance (22). A trial assessing caloric restriction through low glycemic dietary load found that markers of whole body oxidative burden could be improved (23). These benefits are borne out on the cellular level mechanistically throughout the cardiovascular system, and are interwoven with inflammation (24). As such, this may be a valuable frontier for which the quality of ESHP can be increased. Later in this review, we discuss the mechanisms through which caloric restriction could unwind the pathologic state observed during ESHP.

Provided that glucose is a cheap and easy to dissolve substrate that satisfies the minimum nutritional requirements of the heart, it has been a staple of machine perfusion settings. Although, results from our own porcine model utilizing post-prandial glucose and insulin have shown that the heart experiences disturbances in glycolytic pathways which limit energetic efficiency of glucose. During ESHP, glucose uptake increases over time, yet myocardial oxygen consumption declines (13). This is in similarity to heart failure, which features a dependence on glycolysis rather than glucose oxidation, for which fatty acid oxidation becomes inhibited through the production of malonyl-CoA (a potent inhibitor of fatty acid oxidation), representing a loss of metabolic flexibility (25). Following ESHP, the activity of pyruvate kinase, a rate limiting enzyme for flux into the tricarboxylic acid (TCA) cycle, is decreased (13). This suggests that the use of glucose for high energy oxidative phosphorylation is impaired. Concomitantly, there is a transition from glucose oxidation towards non-oxidative pathways such as the pentose phosphate pathway (PPP) and hexose biosynthesis pathways (HBP), evidenced by increased glucose-6-phosphate dehydrogenase (G6PD) activity and protein O-GlcNacylation (markers of flux through the PPP and HBP respectively) during ESHP (16). These results suggest that during ESHP, the heart may experience an energy-deplete state as it diverts metabolism away from glucose oxidation towards combatting oxidative stress. Indeed, reduced glutathione (the heart's main anti-oxidant) is decreased during ESHP alongside induction of other oxidative stress markers, such as malondialdehyde (16), perhaps as a result of perpetual oxidative stress while isolated on the semi-physiologic ESHP platform (26). We showed that provision of pyruvate during late perfusion resulted in a partial reversion in the loss of cardiac index during normothermic ESHP (13), perhaps through a bypass of glycolytic blockages and flux through non-oxidative PPP/HBP pathways to feed directly into glucose oxidation. This is supported by another study comparing glucose and pyruvate supplementation during 6-hour hypothermic ESHP, concluding that pyruvate more efficiently contributed to the TCA cycle in this context (27). Further studies back within the context of normothermic ESHP have shown that functional decline, at least over 4 h of perfusion, can be ameliorated by constant provision of sodium pyruvate with hemofiltration (to prevent ensuing hypernatremia) (28). However, no tissue characteristics were presented (28). These results suggest that a drop in functional output during ESHP, irrespective of temperature, may occur by dysregulation of glycolysis, however even amelioration of functional decline during ESHP does not necessarily preclude tissue damage, oxidative stress, and resultant inflammation that can predispose an individual to primary graft dysfunction (PGD) following transplantation. Therefore, analysis of new metabolic support strategies should consider not only their effectiveness at improving functionality of the organ whilst on the apparatus, but also their ability to quell the inflammatory and oxidative milieu that pervades ESHP.

### Lactate as a non-prognostic marker during ESHP

Lactate was originally touted as a sensitive and specific marker for poor post-transplant outcomes following ESHP (29), and was shown to be higher in runs where the hearts were turned down for transplant (30). Therefore, it made sense to investigate lactate as a potential prognostic marker, since current clinical ESHP apparatuses lack tools to directly measure functional output to prognosticate outcomes specific to the graft. Additionally, lactate has been shown to be predictive of poor prognoses in other clinical settings such as heart failure, myocardial infarction, acute coronary syndrome, and following percutaneous coronary intervention (PCI) (31–33), making it seem appropriate to monitor in this context. However, further studies in the context of ESHP have shown that the predictive value of lactate is limited, in pre-clinical models and both DBD and DCD scenarios. Our laboratory's pre-clinical model showed that lactate failed to correlate with direct measures of functional output during 8 h of ESHP (34). Further clinical studies have verified that this extends to post-transplant outcomes across donation settings. Truby et al. analyzed myocardial metabolites over the course of clinical ESHP, including hearts procured from both DCD and DBD donors, and found that neither end-of-run lactate nor overall change in lactate was associated with PGD post-transplant, after a median perfusion time of 234 min (35). Another study focusing on DCD heart transplantation following ESHP found no difference in arterial lactate profiles between individuals requiring mechanical circulatory support post-transplant (36), with similar median perfusion times as the Truby et al. study.

### A mechanistic point of view on lactate during ESHP

The canonical understanding of lactate is that it is formed as the cell undergoes anaerobic metabolism; in one study of a DCD ESHP model, lactate generation due to anaerobic metabolism was suggested to explain why dilution of perfusate hemoglobin appeared to perform worse over 4 h of perfusion (37). However, it should be noted that the myocardium is known to produce lactate even under perfectly aerobic conditions, and that lactate has been shown to contribute to oxidative metabolism at the mitochondria as much as glucose, especially under loaded conditions (38). A more nuanced view of lactate, the “Lactate Shuttle Theory”, suggests that lactate is highly fluxed intercellularly and acts to improve the efficiency of energy metabolism, as well as act as an energy substrate during states of stress, including ischemia (39). Therefore, the generation of lactate may simply reflect a change in metabolic programming incumbent upon stress endured during ESHP, perhaps more indicative of dynamic myocardial homeostasis than irreversible harm. We know this to occur during ESHP: provided that flux through glycolysis to the TCA cycle may be impaired due to lowered pyruvate kinase activity following 12-h of ESHP (13), it may be of no surprise that lactate is produced (9). Additionally, lactate levels and utilization of lactate by the myocardium can be influenced by the presence of other competing substrates, rather than irreversible pathology to the heart. A lowering of perfusate lactate through consumption by the adapting myocardium may introduce inaccuracy when lactate levels are used to prognosticate outcomes. These properties of lactate may explain why lactate fails to predict negative outcomes in this setting. Cardiac troponin-I may be an obvious alternative to lactate given its routine use in diagnosis of myocardial infarction, however cardiac troponin-I formed during ESHP has not associated well with post-transplant PGD, either (35). An ideal biochemical prognosticator measurable via the perfusate would need to be a more stable agent, formed and released only when the myocardium has undergone irreversible damage. It is possible that micro-RNAs or donor-derived DNA released to the perfusate could fulfill this role (40, 41), however more research is required.

## Potential mechanisms for glucose mediated pathophysiology during ESHP

**Figure 2:**
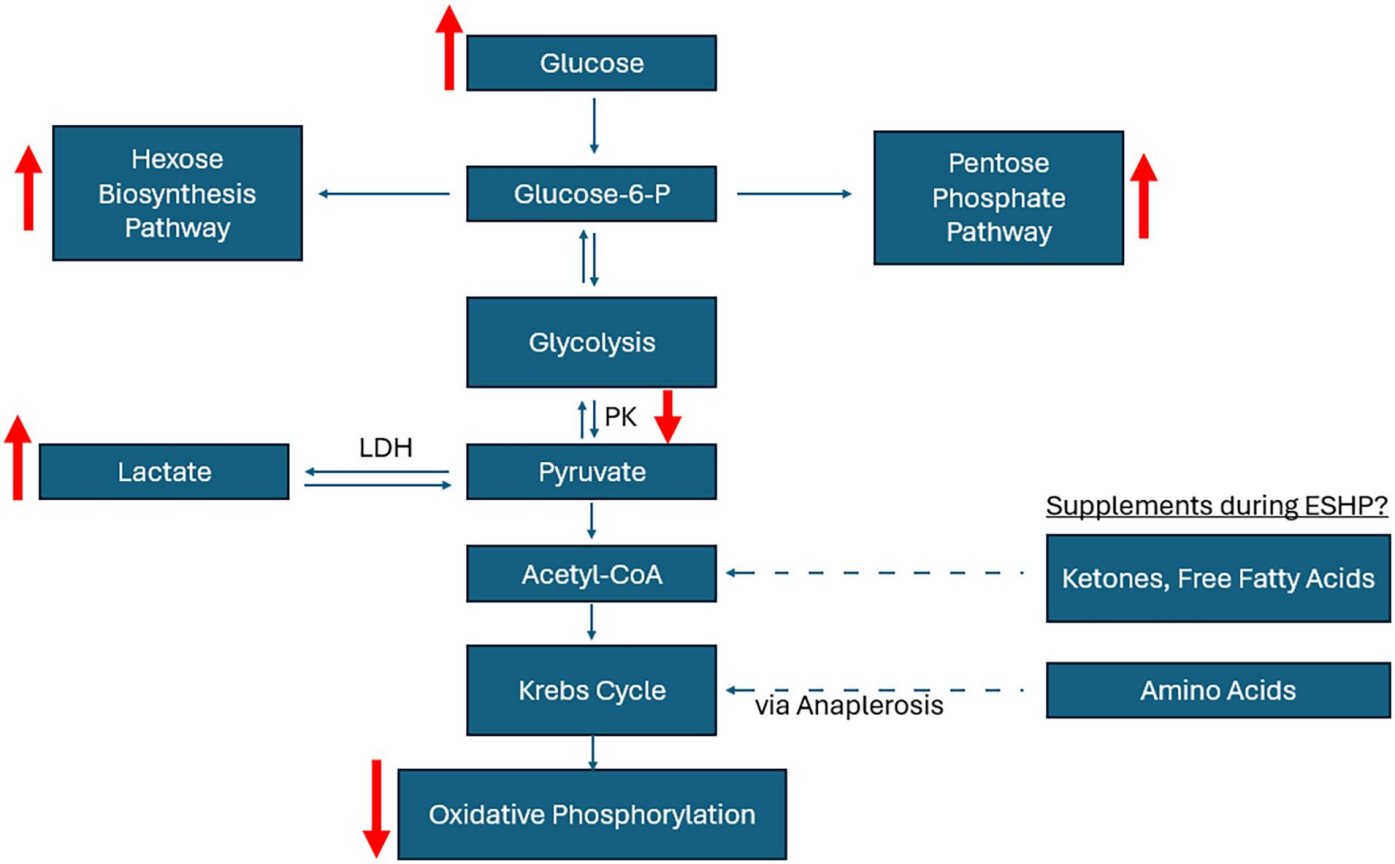
Summary of metabolic observations during ESHP (denoted by red arrows). Elevated glucose through supraphysiologic provision leads to increased flux of glucose through both the hexose biosynthesis and pentose phosphate pathways, worsened by lowered activity of pyruvate kinase during ESHP. Supplements such as amino acids and particularly ketones or free fatty acids may avoid manifestations of changed metabolic flux by providing acetyl-CoA directly to the Krebs Cycle. Acronyms: ESHP, *ex-situ* heart perfusion; LDH, lactose dehydrogenase; P, phosphate; PK, pyruvate kinase.

**Figure 3:**
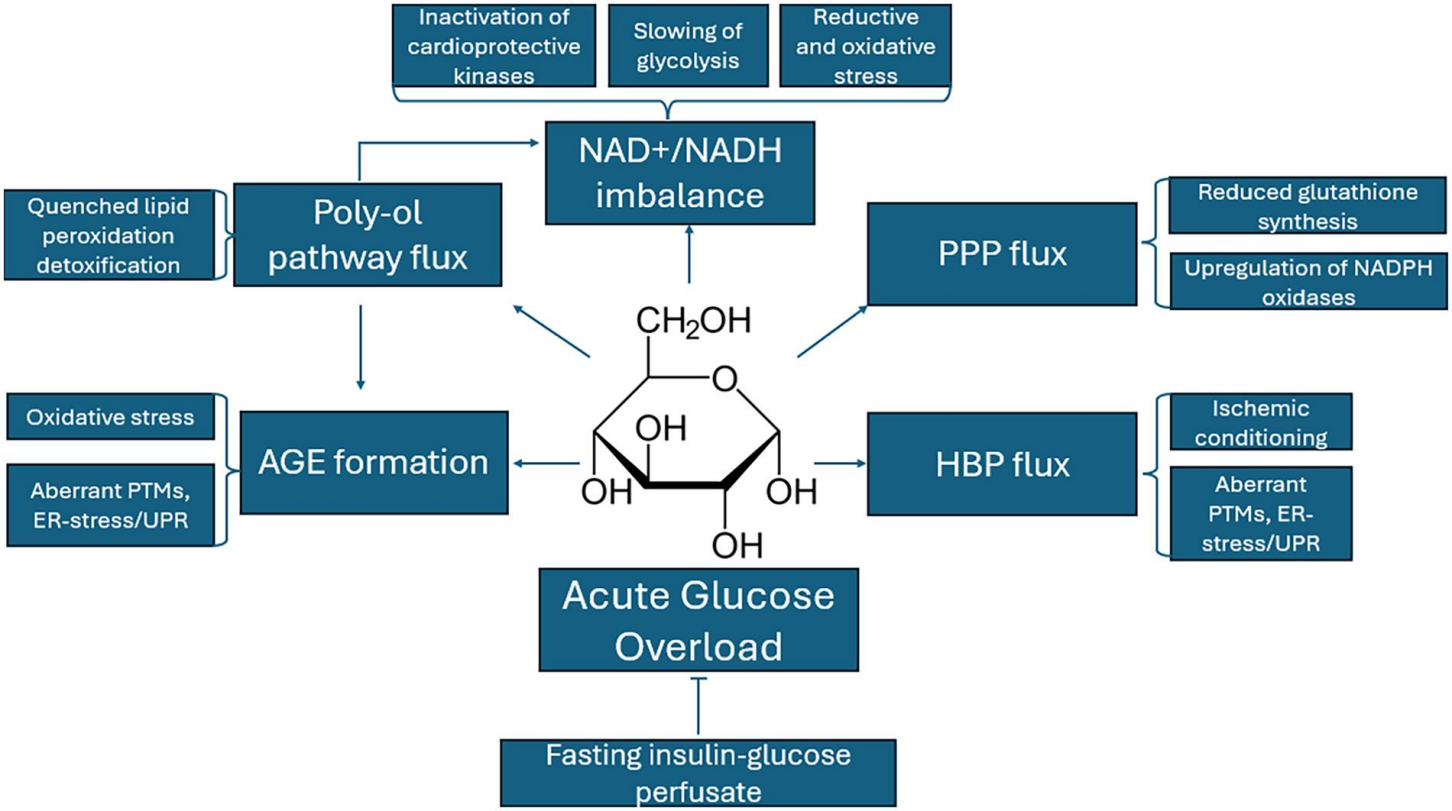
Summary of the potential mechanistic effects of acute glucose overload on the myocardium during ESHP; such harms (or benefits) may be abrogated by glucose restriction during ESHP. Acronyms: AGE, advanced glycation end products; ER, endoplasmic reticulum; HBP, hexose biosynthesis pathway; NAD, nicotinamide adenine dinucleotide; PPP, pentose phosphate pathway; PTM, post-translational modifications; UPR, unfolded protein response.

Beyond the evidence for the dysregulation of glucose processing pathways that occur during ESHP in the context of high glucose and insulin levels, there is an abundance of evidence to suggest that acutely high glucose and insulin levels themselves are associated with induction or exacerbation of oxidative stress (42–44) Acute hyperglycemia and high insulin occurring during critical illness causes damage to various cellular structures, including mitochondria, leading to redox imbalances inside the cell. Cardiac dysfunction observed in patients with critical illness is associated with increased levels of blood glucose and insulin, and the appearance of cardiac dysfunction coincides with increased glucose and insulin levels (insulin resistance) (42–44) Further, ischemia-reperfusion endured before the onset of ESHP has the opportunity to interact with hyperglycemia-induced stressors on the apparatus to worsen the degree of pathology endured (44, 45). An early study showed that hyperglycemia could abolish the positive effects of ischemic preconditioning (46). This has direct implications for ESHP, which entails revitalizing the heart following a period of global ischemia and restabilizing it before another period during implantation. Given the presumably heightened potence of insulin during ESHP and the provision of high levels of glucose, alongside evidence of blockages in glycolysis during ESHP (13), it is likely that there is an overload of glucose to the cytosol. This would force glycolytic flux into alternative biochemical pathways, such as the pentose phosphate pathway (PPP), the hexose biosynthesis pathway (HBP), and the poly-ol pathway (Figure 2). Apart from lacking glycolytic flux for oxidative metabolism, it is possible that acute flux down these alternate pathways during ESHP contributes to the degree of oxidative and endoplasmic reticulum (ER) stress as well as inflammation endured during ESHP. In the following sections, we outline problematic mechanistic associations between glucose metabolism and the pathology that is observed during ESHP (summarized in Figure 3).

### Pentose phosphate pathway

The PPP, depending on how it is regulated, can be either protective or deleterious. While flux through the PPP can be protective by driving increased glutathione synthesis through NADPH production to combat intracellular oxidative stress via glutathione reduction, this same NADPH production could also fuel NADPH oxidases (NOX) (47). In healthy tissue, it is known that in response to oxidative stress, G6PD, the rate limiting enzyme of the PPP, is upregulated, and provides cardioprotection against oxidative species in cardiomyocytes (48). Its depletion from mice exacerbated pathology in models of either myocardial infarction or pressure overload heart failure (49). On the other hand, the NOX enzymes are known to be upregulated during ischemia reperfusion injury (50), and have been implicated in hyperglycemia-exacerbated ischemia reperfusion injury (44). Oxidative stress production by PPP flux and NOX activity has also been demonstrated in models of diabetic cardiomyopathy, whereby acute elevations in glucose have been shown to stimulate NOX recruitment, activation, and cellular oxidative stress in cardiomyocytes (51). During ESHP, it is possible that generated NADPH, in the heart's effort to re-establish redox balance through the generation of reduced glutathione (GSH), has a greater chance of being used by NADPH oxidases to produce superoxide rather than form GSH. This is evidenced during heart failure: G6PD is known to be upregulated during human heart failure, however inhibition of the oxidative PPP in the failing heart either with or without acute hyperglycemia enhanced glucose oxidation and mitigated oxidative stress (52, 53). While the role of NADPH oxidases are yet to be studied in the context of ESHP, which features much more acute timescales than that of the setting of heart failure described here, this prospect appears problematic for glucose as a supportive substrate. It is possible that PPP flux in the context of NOX upregulation contributes to the energetic inefficiency of glucose described during ESHP (27).

### Hexose biosynthesis pathway

Hexose biosynthesis pathway activity is also observed during ESHP, as protein O-GlcNacylation is increased (16). There are conflicting suggestions for the role the HBP plays in the myocardium; some studies have suggested that it is cardioprotective, especially before ischemia-reperfusion injury (54–56), and others that have suggested it to be pathological through aberrant post-translational modifications to myocardial proteins (57–59) As examples of aberrant modification, O-GlcNacylation of respiratory complex proteins in the mitochondria (complexes I, III and IV) under hyperglycemic conditions reduced mitochondrial function and decreased cardiomyocyte ATP levels (60). An additional study showed that O-GlcNacylation decreased cardiac mitochondrial membrane potential, and therefore ATP generation capacity, through modulation of DRP1, a regulator of mitochondrial remodeling (61). While it seems on the whole that protein O-GlcNacylation during acute settings is protective, whereas chronically increased O-GlcNacylation leads to metabolic dysregulation (57), acute cardioprotection via O-GlcNacylation may not always be the case. For example, it has been shown that HBP activity over the course of a 2–6 h infusion of glucose leads to modification of proteins involved in insulin sensitivity (e.g., IRS1/2, AKT, and GLUT4), leading to resistance to glucose uptake (62). Acutely high glucose has also been shown to O-GlcNacylate CaMKIIδ (a calcium activated kinase implicated in damage due to ischemia-reperfusion injury (63), which led to its activation and increased production of reactive oxygen species (ROS) in cardiomyocytes (59). Thus, the flux of glucose through this pathway during ESHP may be an adaptive, protective response by cardiomyocytes to survive ischemia-reperfusion injury (perhaps through mitochondrial transition pore stabilization and inhibition of calcium overload (64) that after re-stabilization interferes with the normal functioning of proteins in the cytosol and mitochondria. We have noted that during ESHP, there is induction of ER stress and the unfolded protein response alongside observations of increased O-GlcNacylation, suggesting this may be a response of the myocardium. In support of this association, other studies have shown that the HBP becomes upregulated upon ER stress and that O-GlcNacylation can reduce ER-stress mediated cardiomyocyte death (65). Further study in the context of ESHP is required: it would be of interest to upregulate the HBP (perhaps by supplementation of glucosamine) to utilize its effects on improving mitochondrial resilience during stress conditions, if it can be demonstrated not to provoke widespread cellular dysfunction.

### Poly-ol pathway

**Figure 4:**
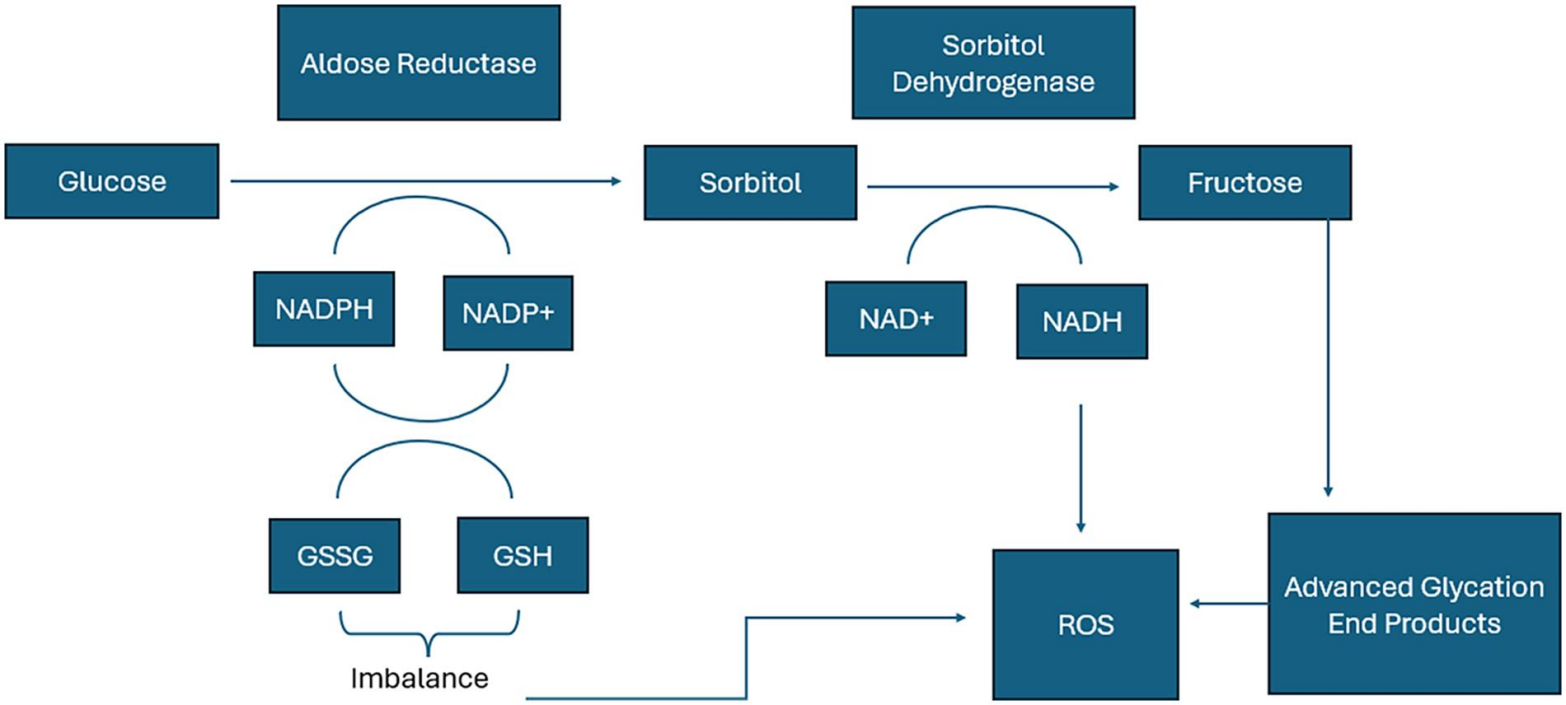
Contribution of the poly-ol pathway to disturbances in NADPH, GSH, and NAD + levels and production of advanced glycation end products (AGEs). Flux of glucose through the poly-ol pathway consumes NADPH available for reduction of glutathione, and produces NADH, which may contribute to reactive oxygen species (ROS) formation via NADH oxidases. Fructose, as a byproduct of glucose metabolism by the poly-ol pathway, is a known contributor to the formation of advanced glycation end products, which promote ROS formation and ER stress. Acronyms: AGE, advanced glycation end products; ER, endoplasmic reticulum; GSH, glutathione; NAD+, nicotinamide adenine dinucleotide (oxidized form); NADH, nicotinamide adenine dinucleotide (reduced form); NADPH, nicotinamide adenine dinucleotide phosphate (reduced form); ROS, reactive oxygen species.

The poly-ol pathway (Figure 4) is another alternative glucose pathway that increases in flux during states of hyperglycemia. As a whole, the poly-ol pathway transmutes glucose into sorbitol, then fructose while producing NADH and consuming NAD^+^ and NADPH (66). Therefore, increased flux through the poly-ol pathway due to hyperglycemic conditions during ESHP can mediate redox imbalance by consuming NADPH needed for glutathione reduction. There is also a risk of increased redox stress by imbalance of the NADH: NAD^+^ ratio, for which excessive NADH is known to manifest as first a reductive stress followed by oxidative stress when NADH oxidation begins to yield high amounts of superoxide at complex I (67, 68). Indeed, cells cultured under hyperglycemic conditions showed aldose reductase-dependent increases in the generation of oxidative species (69). Further, a physiologic role of aldose reductase is to mediate detoxification of lipid peroxidation byproducts, such as 4-hydroxynonenal (4-HNE), which are highly reactive and even form mutative adducts with DNA (70): an inundation of this enzyme with glucose during ESHP may prevent it from handling lipid peroxidation species. A final potentially pathologic aspect of poly-ol pathway flux is that it yields high amounts of fructose, which is a more potent substrate for non-enzymatic modification of proteins with advanced glycation end products (AGE) (71). As described below, AGE can increase the oxidative stress burden during ESHP. In support of the poly-ol pathway's role in AGE formation, inhibition of aldose reductase mediated lowered burden of AGE in rat lens (72).

### Advanced glycation end products

As alluded, hyperglycemia during ESHP also predisposes tissue to the formation of AGE, independently of enzymatic reactions. Termed Maillard reactions, amino groups on protein side chains such as lysine and arginine can react with carbonyl groups of sugars such as glucose and ribose, which undergo further reactions when exposed to oxidative stress to create reactive dicarbonyl compounds, such as methylglyoxal, glycoxal, and glucosone, which react with amino groups on proteins to create AGEs (73). These can be formed in the presence or absence of hyperglycemia. However, during states of oxidative stress and hyperglycemia, there is an accumulation of AGE within the cytosol that mediates cellular dysfunction (74). AGEs crosslink amino groups within and between proteins, yielding abnormalities in protein folding and polymerization of proteins, abrogating their function as well as triggering the unfolded protein response, inflammation, and ER stress. There are also receptors for AGE (RAGE), which when bound by AGE trigger signalling cascades that result in production of inflammatory cytokines and exacerbation of oxidative stress, as well as signal for the induction of the unfolded protein response (75). Provided that AGE are formed from interaction with oxidative species, yet also contribute to the development of oxidative stress, this constitutes a positive feedback loop that amplifies damage to the tissue and the inflammatory response (74). Therefore, AGE may play an important role in the ER stress, inflammation, and oxidative stress endured during ESHP, provided that oxidative stress endured from ischemia-reperfusion injury can begin this positive feedback loop, which may become untenable for the heart over the course of perfusion.

## The potential benefits of glucose restriction during ESHP

On the basis of the aforementioned mechanistic insights, high glucose and insulin as the standard substrate provision approach during ESHP may not provide an efficient and cardioprotective metabolic outlay for the donor heart, and may instead contribute to functional decline via aberrant glucose flux and non-enzymatic reactions. Thus, avoiding flux down these pathways through glucose restriction would be anticipated to improve energy-yielding metabolism and perhaps decrease oxidative stress generation during ESHP.

Recent evidence from human and animal studies suggests a strong association between low-caloric intake (particularly low glucose) and lower oxidative damage to cellular organelles, particularly mitochondria, and improved cardiovascular health resulting in enhanced longevity (76). The factor linking glucose consumption, oxidative stress, aging, and cardiac function may proceed through avoiding the abovementioned mechanisms. In the context of ESHP, we have previously shown that during ESHP, perfusion in the working mode, whereby the ventricle is loaded and must act against pressure, is associated with a variety of beneficial effects in comparison to non-working mode (coronary perfusion only): in working mode vs. non-working mode, we have shown that there is reduced levels of protein carbonylation and malondialdehyde, reduced expression of inflammatory markers such as TNF-alpha, IL-1B, and NfKB-p65, and lowered activation of ER stress pathways (16, 17). The beneficial effects of WM perfusion may proceed through a relative nutrient deprivation of the myocardium in comparison to NWM, as there was increased activation of AMPK, a cellular nutrient sensor which also has protective roles when oxidative stress is endured (16). During WM perfusion, the necessity for greater ATP generation to fuel loaded contraction may induce a state of relative nutrient deprivation in the myocardium, manifesting the positive effects of caloric restriction.

### The potential role of NAD as a mediator within nutrient restriction

In recent years, novel evidence has strongly suggested that oxidized nicotinamide adenine dinucleotide (NAD^+^) can play an additive role in tissue protection during fasting states. NAD^+^/NADH ratio regulates the formation of oxidative stress, intracellular ATP production, and metabolic integrity. While higher NAD + is associated with improved energy-producing metabolism and stress resistance, low NAD^+^ is linked to aging, oxidative damage, and cardiac dysfunction (77). Fasting and low glucose availability increase NAD^+^ levels and AMPK expression and activity, and increasing NAD^+^ levels have been shown to alleviate cardiac dysfunction in pressure overload models (78). These effects of NAD^+^ are believed to take place by promoting various cytoprotective mediators, including the sirtuins (79, 80). Sirtuins are a family of seven highly conserved NAD^+^-dependent deacetylases that act as cellular sensors and regulate metabolic processes (81). Among those, Sirtuin-3 is known to protect the heart from oxidative stress and related damage (82). Sirtuin-1 and its downstream mediator peroxisome proliferator-activated receptor gamma coactivator 1-alpha (PGC-1α) have been shown to exert striking cardioprotective effects in a broad range of experimental models (83). Besides its diverse cardioprotective effects, AMPK also acts as an upstream enzyme to stimulate Sirt-1 activity through modulation of intracellular NAD^+^ concentration (84).

### Autophagy as a potentially cardioprotective process during ESHP

Along this line, autophagy, which serves to recycle damaged cellular organelles (particularly mitochondria, dubbed mitophagy), especially during stress conditions, could also contribute to cardioprotection during ESHP. The activation of AMPK along with Sirt-1 and PGC-1α suppress mTORC1. Suppression of mTORC1 is associated with improved cardiomyocyte survival and enhancement of autophagy, a lysosome-dependent pathway that clears the cytosol of dysfunctional organelles and misfolded proteins that exacerbate oxidative and endoplasmic reticulum stress (85). Restriction of glucose during ESHP would be anticipated to improve preservation of the heart through increasing the activity of AMPK, sirtuins, PGC-1α, and NAD^+^ as well as suppression of mTORC1 to enhance autophagy (86), culminating in improved longevity of myocardial preservation. Though, while autophagy may enable short term compensation for energy demands, excessive autophagy (linked to PI3K-AKT signalling) due to a perpetually low energy state may instead exacerbate functional decline, inflammation, and oxidative stress by depleting necessary organelles for energy production and redox balance (86). Since function was better preserved in working mode, featuring both AMPK and AKT activation, it is possible that autophagy may be cardioprotective or cardiotoxic during ESHP. More study is required. Given that autophagy is highly regulated by the availability of nutrients to the cell, it may constitute a mechanism targetable by adequate substrate provision through which metabolism, inflammation, and oxidative stress can be better addressed. Autophagy has not been previously characterized in the context of ESHP.

With reference to lactate, canonically understood to play the role of regenerating NAD + during ischemia such that anaerobic glycolysis can continue, nonetheless produces NAD + which may have beneficial effects on the myocardium through the abovementioned mechanisms. Provided that the myocardium during ESHP is likely to have a high NADH:NAD + ratio from flux of glucose through the poly-ol pathway, the generation of NAD + via lactate generation may represent an effort by the myocardium to effect homeostasis of this ratio. Further study of the role of NAD + during ESHP and the contribution of lactate is required, however.

## Alternative substrates & supplements during ESHP

While glucose restriction could have enormous benefits to the immuno-oxidative milieu that appears to pervade ESHP, these benefits may only be borne at low concentrations of glucose, whereas the metabolically active heart requires oxidizable substrate to satisfy ATP generation requirements. In the following sections, we discuss alternatives that may be explored for ESHP to satisfy or facilitate myocardial requirements.

### Freefatty acids as an energy providing substrate during ESHP

Free fatty acids (FFAs) are the heart's preferred substrate *in vivo* (25), comprising 70%–80% of the heart's metabolic fuel *in vivo*. However, there are currently no studies evaluating whether supplementation of FFAs can ameliorate functional decline during ESHP. We have shown previously that during ESHP, there is a swift consumption of almost all available non-esterified fatty acids in solution by the heart by 5 h post-reperfusion, however there is a concomitant increase in perfusate triglyceride content (13). This may be due to inhibition of lipoprotein lipase by heparin (87), an anticoagulant used during ESHP. Nonetheless, the heart may reach a state of metabolic inefficiency by this timepoint, leading to functional decline as it begins to rely solely on dysregulated glycolysis to feed the TCA cycle. We have noted that leading up to 5 h post-reperfusion during ESHP is when the most severe functional decline occurs, suggesting that a metabolic switch from fatty acids to glucose may predispose the heart to energy shortage. This is reasonable given that beta oxidation of fatty acids provides a surplus of reduced electron carriers (NADH and FADH_2_) and acetyl-CoA for the TCA cycle to support oxidative phosphorylation, independently of glycolytic metabolism. This may also impact redox balance within the heart: IntraLipid (IL), a clinically approved lipid emulsion, was tested in a porcine DCD model in combination with ESHP. Administration of IL significantly reduced markers of oxidative stress associated with ESHP and increased inotropy following reperfusion, though this effect was thought to be due to induction of post-ischemic conditioning rather than being a substrate mediated effect (88).

### Ketone bodies as an energy providing substrate during ESHP

Ketone bodies (KBs) are produced by the liver when glycolytic substrates are extremely low within the body to ensure that energy homeostasis is maintained. Though KBs typically only account for ∼10% of the heart's metabolism *in vivo*, they are readily taken up and metabolized, especially in some forms of heart failure (89–91) KB supplementation has garnered significant attention as a potential treatment for heart failure with reduced ejection fraction (HFrEF), with many clinical trials now evaluating the effects of KB supplementation on the disease (92). In HFrEF, the heart is known to have impaired FAO and glucose oxidation depending on the etiology (25), mirroring our own observations during prolonged ESHP. Metabolism of KBs directly provides acetyl-CoA for the TCA cycle, bypassing the dysfunctional pathways to provide energetic substrate to the energy deprived heart, helping ameliorate functional loss. It has also been suggested that ketone body supplementation can optimize myocardial metabolism by either replacing missing fuel sources or outcompeting pathological metabolism (89, 92). Ketone body administration has also been demonstrated to attenuate inflammation by inhibition of the NLRP3 inflammasome (93), which may also be upregulated during prolonged ESHP given the increase in IL-1B and IL-18 over the course of perfusion (16). It has also been demonstrated that mitochondrial damage in aging hearts can be lessened by ketone body administration, through restoration of mitophagy, leading to increases in cardiac function in diabetic mice (94, 95). Restoration of mitophagy was also associated with resistance to oxidative stress, suggesting that regulation of mitochondria through ketone body administration can also improve redox balance (95). It has been shown in clinical settings that ketones, such as beta-hydroxybutyrate, are avidly consumed by the myocardium during ESHP (35). Given that hearts subjected to ESHP suffer from an inflammatory, oxidative milieu, these results suggest that supplementation during ESHP can mediate benefit through these mechanisms, on top of their utility as an ancillary fuel.

### Amino acids as a substrate during ESHP

Amino acids are used by the myocardium, however to a small degree, making up <5% of the ATP generation in the healthy heart (96). Provided this small contribution to the total amount of ATP requirements by the heart, amino acids are perhaps better utilized as supplements to other oxidative metabolites, preventing them from acting alone as the sole substrate for myocardial contraction. Truby et al. showed that over the course of clinical ESHP, there was a high degree of consumption of amino acids, particularly leucine/isoleucine, aspartate/aparagine, and glutamate/glutamine (35). This may reflect a consumption of anaplerotic substrates during recovery from ischemia, for which amino acids are taken up by the myocardium to replenish TCA cycle metabolites. Glutamate and glutamine (converted to glutamate via transamination) can be converted into alpha-ketoglutarate, providing anaplerotic substrate that may be depleted following ischemia. Beyond anaplerosis, alpha-ketoglutarate has been suggested to contribute to antioxidant defense by upregulation of antioxidant enzymes, on top of directly neutralizing ROS. It can also improve the handling of reactive nitrogen species by combatting the accumulation of resulting ammonia through generation of glutamate (97). As a convergent metabolite of glutamate and glutamine, it may be of more utility to instead supplement alpha-ketoglutarate directly during ESHP. Aspartate and asparagine can also function to improve anaplerosis by being converted into oxaloacetate. Glutamate is also a precursor, alongside cysteine and glycine, for glutathione, required for cytosolic antioxidant defence.

The consumption of these amino acids during ESHP suggests that supplementation could yield greater preservation efficacy. Indeed, proprietary solutions for ESHP mixed with donor blood already contain some amino acids (branched chain amino acids, BCAA), but their role during ESHP is not yet clear. Studies have shown that both supplementing amino acids (particularly, glutamate and glutamine) before periods of ischemia (56, 98) and after periods of ischemia-reperfusion (99–101) improves cardioprotection, suggesting their utility during ESHP to improve pre- and post-ischemic conditioning, and perhaps facilitate recovery on the device. As previously mentioned, glutamine can also be utilized by the HBP to effect protein O'GlcNacylation (56), which may serve as a cardioprotective modification. Taurine may also be of interest to supplement: it has been shown that taurine at early reperfusion can reduce the effects of ischemia-reperfusion injury in isolated rat heart perfusion (102), with a variety of suggested benefits related to calcium ion homeostasis and antioxidant support (namely in reducing byproducts of lipid peroxidation) (103). Normally, BCAAs such as leucine/isoleucine and valine also contribute to the TCA cycle by providing acetyl-CoA and succinyl-CoA, suggesting that they can also contribute to anaplerosis; however, it has also been proposed that high levels of BCAAs can increase mTOR signalling and insulin resistance, which contributes to development of hypertrophy in chronic scenarios (104), with insulin resistance particularly due to metabolism of BCAAs to branched-chain keto acids (BCKAs) (105). Whether this occurs during an acute setting from simple supplementation is not clear. Further research is necessary to investigate the effects of amino acids of varying types during an acute setting such as ESHP.

## Summary of outstanding questions

Provided this discussion of insights into the deleterious effects of high glucose provision or the benefits of its restriction, there appears to be ample opportunity for continued investigation of these mechanisms within the context of ESHP. Based on these insights, we hypothesize that an approach that restricted glucose during machine perfusion would reduce the potentially deleterious effects of elevated glucose flux down alternative pathways such as the PPP, HBP, and poly-ol pathways, as well as the propensity for AGEs to be formed. The relative contributions of each of these pathways during ESHP is yet to be understood fully. We surmise that non-physiologic flux down such pathways contributes to the degree of oxidative and inflammation endured by the heart, particularly from the poly-ol and AGE pathways, given their propensity to establish positive feedback loops that upset redox balance. Further, the potential for the HBP or PPP pathways to potentially contribute to respective post-ischemic conditioning or antioxidant support outside of their potentially pathologic mechanisms are yet to be explored. With further investigation, modulation of these pathways towards their positive functions may prove to be valuable modalities for intervention during machine perfusion. As discussed, the benefits of glucose restriction may not only come from cessation of these potential pathologic mechanisms: directly reducing oxidative stress development and further benefits via downstream signalling mediated by high NAD+/NADH ratio could lead to improved organ preservation. As evidenced by the beneficial effects of WM perfusion, we hypothesize that an elevated NAD + ratio that could be achieved through direct supplementation or caloric restriction could act to mediate cardioprotection through the activation of proteins involved in mitophagy, AMPK, or the sirtuins. It remains to be seen what the contributions of each of these mediators are during ESHP. Further, the replacement of glucose with alternative substrates that play into both redox balance and pre/post-ischemic conditioning may also improve organ preservation, but require individual investigation. The amelioration of oxidative stress, inflammation, and metabolic dysregulation resultant during ESHP provides a rational basis for designing a more optimal substrate provision strategy in the light of a near infinite number of supplements to investigate.

## Conclusion

Current protocols for metabolic support during ESHP invariably utilize glucose, which has a variety of concerning mechanistic properties, especially when provisioned at current levels used clinically. Including restriction of glucose, future metabolic support strategies must explore new substrates and approaches for use during ESHP to accommodate the heart, which exists in a post-ischemic state on the apparatus and is obligated to endure another period of global ischemia before implantation. In addition to reducing the current contributions of metabolism during ESHP to endured oxidative stress, this represents opportunities to tailor support strategies for both post- and pre-ischemic conditioning. While classical research into myocardial metabolism provides helpful insights into how such substrates should behave in the myocardium in models of the ischemic and failing heart, the acute setting of ESHP and the need for continued preservation within the ESHP environment following initial ischemic insult upon procurement diverges from many studies conducted thus far. Optimized metabolic support strategies investigated in the context of ESHP can drive improvements in preservation efficacy and length, for which mechanistic insights obtained from this frontier of research may be applicable to other solid organs preserved by machine perfusion.

## Glossary

AGE: advanced glycation end products
AIF: apoptosis-inducing factor
AKT: protein kinase B
AMPK: AMP-activated protein kinase
ATP: adenosine triphosphate
BCAA: branched-chain amino acids
BCKA: branched-chain keto acids
CaMKII: calcium/calmodulin-dependent protein kinase II
CHOP: CCAAT-enhancer-binding protein homologous protein
DBD: donation after brain death
DCD: donation after circulatory death
dP/dT: derivative of pressure over time
DRP1: dynamin-related protein 1
EC: extracorporeal circulation
ER: endoplasmic reticulum
ESHP: *ex-situ* heart perfusion
FAO: fatty acid oxidation
FFA: free fatty acids
G6PD: glucose-6-phosphate dehydrogenase
GSH: reduced glutathione
HBP: hexose biosynthesis pathway
HFrEF: heart failure with reduced ejection fraction
IL: intraLipid (in one context) interleukin (in another)
IRS: insulin receptor substrate
IU: international units
KB: ketone bodies
MDA: malondialdehyde
mTORC1: mechanistic target of rapamycin complex 1
NAD+: nicotinamide adenine dinucleotide (oxidized form)
NADH: nicotinamide adenine dinucleotide (reduced form)
NADPH: nicotinamide adenine dinucleotide phosphate (reduced form)
NF-κB: nuclear factor kappa-light-chain-enhancer of activated B cells
NOX: NADPH oxidase
NWM: non-working mode
OCS: organ care system
PCI: percutaneous coronary intervention
PGC-1α: peroxisome proliferator-activated receptor gamma coactivator 1-alpha
PGD: primary graft dysfunction
PPP: pentose phosphate pathway
RAGE: receptor for advanced glycation end products
ROS: reactive oxygen species
SIRT: sirtuin
TCA: tricarboxylic acid (cycle)
TNF-α: tumor necrosis factor alpha
TUNEL: terminal deoxynucleotidyl transferase dUTP nick end labeling
UNOS: united network for organ sharing
WM: working mode.
